# Study of Live Lecture Attendance, Student Perceptions and Expectations

**DOI:** 10.1007/s40670-021-01236-8

**Published:** 2021-02-23

**Authors:** Johnathan Emahiser, John Nguyen, Cheryl Vanier, Amina Sadik

**Affiliations:** 1 Third Year Medical Student (OMSIII), College of Osteopathic Medicine, Touro University Nevada, Henderson, NV USA; 2 Chief Research Officer, Department of Research, Institutional Review Board (IRB) Chair, Touro University Nevada, Henderson, NV USA; 3 Amina Sadik, Basic Sciences Department, College of Osteopathic Medicine, Touro University Nevada, NV Henderson, USA

**Keywords:** Attendance, Pre-clinical, Medical student, Lecture, Active learning, Undergraduate medical education

## Abstract

Declining lecture attendance has been an ongoing concern for educators involved in undergraduate medical education. A survey was developed (a) to gain insight into the reasons students skipped class, (b) to identify the type of study materials they were using, and (c) to determine what they thought would motivate them to come to class. The survey was sent to 317 first-year and second-year medical students, and 145 (45%) responded. Only 63% of first-year students and 53% of second-year students attended any lectures that were not mandatory. The attendance was higher for students who aspired to less competitive specialties such as pediatrics and family medicine. The most popular reasons for not coming to class were related to the efficiency of information intake and instructor or class style. The most heavily used resources (> 60%) were materials or recorded lectures provided by the instructor. The second-year students also heavily used outside study materials for Board exams, such as Pathoma (50%). Students’ ideas for what might increase their attendance suggest that they perceive that the lectures may not prepare them for Board exams, and they would like faculty to address Board related content more often in class and on assessments. Respondents also suggested that teaching practices might be improved through faculty development. Faculty awareness of and references to Board exam content, embedded in strong teaching practices, may help students find more value in live lectures. Carefully designed active learning sessions may change students’ minds regarding the relevance and value of these sessions.

**Supplementary Information:**

The online version contains supplementary material available at 10.1007/s40670-021-01236-8.

## Introduction

Lecture attendance has been a growing concern for medical educators for over a decade [1], yet the downward trend continues. From 2015 to 2017, the percent of second-year medical students who reported attending in-person courses of lectures “often” or “most of the time” declined from 52.3 to 47.3% [2]. Poor lecture attendance can negatively affect the medical school experience in several ways. For example, faculty morale is damaged by low lecture attendance, which can erode the quality of instruction [3] and faculty retention rates. In addition, students who do not attend class may not get appropriately socialized into the professional behaviors and habits that are expected after graduation [3]. Finally, students who do not attend classes regularly may have poorer academic outcomes than peers who attend classes diligently [4], but see [5, 6].

The reason for the downward trend in lecture attendance has several explanations, with most rooted in evolving educational technology. First, most medical schools currently record lectures, which allows students to view them at their own pace—accelerating or rewinding the recordings as needed. Studies have not always found a link between recording lectures and declining attendance [7, 8], however, because some students may view them instead of attending the live class session, thereby decreasing attendance, while others may use them to review selected live lecture material, which may have been unclear [9]. A second contributor to low-class attendance is the growing number of supplemental medical education materials, some of which are freely available online. Favored for the concise and "high-yield" focus on USMLE Step 1 and COMLEX-USA Level 1, at least 90% of medical students use online resources to study [9].

Because recorded lectures and supplemental educational materials have only recently become widely available, there is limited information about how different groups (e.g., genders, ages) choose to spend their limited time for medical education. For example, a recent study from Australia suggested that there may be some age-related differences in the use of educational resources, but no differences due to gender [9]. In another work, females were more likely to attend live lectures, and first-year medical students attended lectures more often than second-year medical students [7]. Results among studies may be inconsistent partly because the psychological attributes of students who attend class may regularly differ from students who do not. For example, a study of second-year medical students in a pathophysiology course found that students who did not attend class regularly had higher levels of self-efficacy and self-regulation [5].

Attendance rates at live class sessions, especially when the class sessions are recorded and available online, is also related to the teaching style adopted by the instructor. Students, particularly males, attending class sessions that consisted entirely of lecturing reported difficulty concentrating [10]. When the material is entirely presented in a lecture format, there is evidence that students who prefer to watch online videos may perform as well or better than those who attend class [11]. Students who attended live lectures as opposed to watching the same lectures which had been recorded and made available online performed equally well in the course [12, 13]. If a lecture had a strong basic science research context (as opposed to an applied basic science context), students performed better if they used lecture capture [13]. Replacing lectures with more active learning has been proposed as a method to increase attendance [10].

The concern about lecture attendance is often framed by faculty concerns about students missing class. Faculty tend to view class time as an opportunity to help their students develop their professionalism, critical thinking skills, and deeper understanding of the sciences that are useful for future physicians. In contrast, students view class time as one of many alternative venues in which to learn facts [3]. Since there is no definitive relationship between class attendance and grades [4, 6], in the students’ view, class attendance may not be the best use of their time, especially when it comes to preparing for the Board exams. Consequently, professors’ endeavors in their educational role is often at odds with rote memorization required for licensing examinations in the era of “step 1 climate” [14]

Very few studies (e.g., [7]) have tried to probe in more detail the students’ perceptions, perspectives, and motivations for attending class. There is a need for a greater understanding of why students do not find class attendance useful, what they do instead of going to class, and what the students themselves think might bring them back to the lecture hall.

We surveyed first-year and second-year medical students at Touro University Nevada College of Osteopathic Medicine (TUNCOM) to learn about:

- The rationale behind students' decisions to skip class
- Students’ use of materials to engage in medical education
- Students’ view of the school curriculum to prepare them for Board examinations and clinical rounds
- How to motivate students to attend class more regularly

We hypothesized that more active learning approaches and better cognitive integration across the curriculum might motivate students to attend class more often.

## Study Design and Methods

The TUN Institutional Review Board (IRB) determined that this project was exempt from further oversight, as data was collected as part of an educational quality improvement endeavor. An electronic survey was designed using SurveyMonkey Software for first and second-year medical students at TUNCOM (Table S1). The survey was comprised of questions about the following topics:

- Frequency of class attendance, study activities before and while attending class
- Reasons for missing class, resources used instead of attending class, and motivations to attend class, such as professor performance, curriculum/learning session structure, and classroom environment
- Student confidence in the curriculum to prepare them for Board examinations, evidence-based medicine, and matching into residency
- Student learning styles, and approaches to improve study habits, preparation for tests, and stress management
- Demographic information

Questions either asked students to choose one answer on a Likert scale, to rank all appropriate choices in order of importance, or to select all options that applied (Table S1). When necessary, responses were coded. Resources used to prepare for the class were ranked by respondents, and the top three from each participant were included for analysis. Multiple response questions were summarized by computing the percentage indicating a response and associated confidence intervals [15]. The face and content validity of the survey was established by students and faculty. Eleven medical student volunteers (first-year and second-year) reviewed the survey questions. Changes were made according to the comments and suggestions, and a second review was conducted by the same group. Student volunteers were given ten dollar gift cards as an incentive for participation in both sessions. The resulting survey questions were then reviewed by two survey research experts who were not part of the research project for a final validation step.

In May 2019, the survey was launched via email to all first-year and second-year medical students (*n* = 317) at TUNCOM. Only one response per email address was allowed. The survey was prefaced with a consent form acknowledging that all answers would remain anonymous. As an incentive, students were given the choice to be entered to win a $200 gift card after completing the survey if they provided their names at the end of the survey. The names were not linked with the data during the analysis. The responses were gathered for two weeks after the initial email, during which three reminders were sent. Most surveys were complete; eight responses out of 19,285 possible answers were missing (0.04%). Analyses involving missing values were computed with the missing value(s) dropped.

For summary statistics, responses were summarized by calculating proportions and estimating 95% confidence intervals. When of interest, group comparisons were made using logistic regressions because the questions were designed to evoke yes/no responses. Specifically, we looked for an association between class attendance and learning style or confidence that classes were providing preparation for boards and evidence-based practice. There were some questions that required a response that was not dichotomous. For example, class attendance was dichotomized into two groups: those who attended at least some lectures voluntarily (1), and those who attended only mandatory lectures (0) because the latter group was quite large, and the groups for those who attended at least some lectures voluntarily were not large enough to support analysis separately, so they were combined. The questions about confidence in Board preparation and evidence-based medicine were dichotomized to split those who were confident or very confident from those who were somewhat confident, not confident, or could not judge. Effect sizes were shown using odds ratios and 95% confidence intervals. The analyses were completed using Rv3.5.1 software (www.R-project.org) and the “car” package [16].

## Results

Of 317 surveys sent, 145 students responded (45% response rate), consisting of 63.4% first-year students and 36.6% second-year students. The two groups were similar in GPA and gender (Table 1), and the gender representation was statistically the same as medical students (MD and DO) nationwide in 2018–2019 (DO students 52% female of 8,442 students, [17] MD students 49% female of 21,622 students [18]; Fisher exact test comparing gender distribution between MD/DO students overall and study respondents pooled between years *p* = 0.06) and for the first year class (44% female of 187 students, Fisher exact *p* = 0.91). Still, there were differences in preference for specialty, with more first-year respondents preferring internal medicine, family medicine, and pediatrics, whereas second-year respondents had a stronger preference for psychiatry (Table 1). Although both first-year and second-year medical students identified making a contribution to society and helping others as the primary motivators to enter the medical field, there were also some differences between classes. First-year students were more likely to cite family or community expectations compared to their second-year counterparts. In contrast, second-year students were more likely to answer self-worth as a reason for entering medicine compared to the first-year respondents (Table 1).

**Table 1:** Demographic information for survey respondents by program year. Grade point average = GPA

		Year 1	Year 2
Number of respondents		92	53
GPA	2.0–2.5	9%	2%
	2.5–3.0	18%	17%
	3.0–3.5	51%	65%
	3.5–4.0	22%	15%
Gender	% female	43%	42%
Specialty (within top 3)*			
	Internal Medicine	48%	36%
	Emergency Medicine	30%	38%
	Family Medicine	32%	19%
	Unsure	18%	25%
	Surgery	22%	17%
	Anesthesiology	18%	17%
	Physical Medicine and Rehabilitation	11%	19%
	Psychiatry	8%	21%
	Neurology	10%	13%
	Obstetrics and Gynecology	10%	11%
	Gastroenterology	7%	15%
	Pediatrics	16%	6%
	Orthopedic Surgery	11%	9%
	Radiology	5%	11%
Reason to enter the medical field			
	Contribution to society	73%	66%
	Helping others	72%	70%
	Self-worth	24%	36%
	Serve the medically indigent	18%	17%
	High income	14%	23%
	Family/Community Expectations	18%	8%
	Professional prestige	7%	9%
Lecture attendance			
	Every day	26%	15%
	Attended most lectures	20%	19%
	Attended half of the lectures	17%	19%
	Attended mandatory lectures only	37%	47%

### Patterns of Live Lecture Attendance

**Fig. 1:**
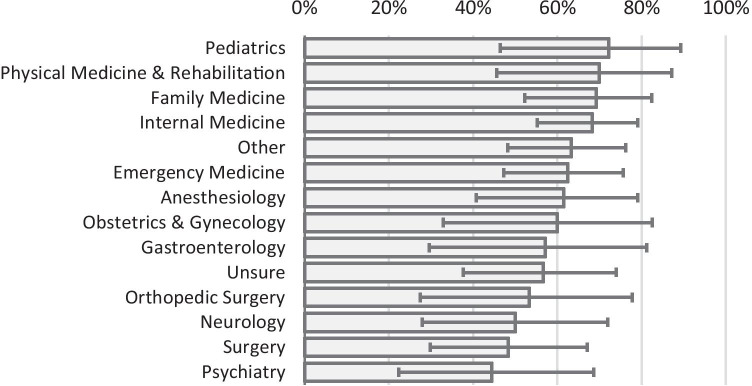
Percent (95% confidence intervals) of students who attended some or all non-mandatory lectures by intended specialty. Across all students, the mean attendance of some or all non-mandatory lectures was 61%

In both cohorts, more students attended only mandatory lectures than attended lectures every day (Table 1). Second-year students reported attending non-mandatory lectures less than first-year students (53% vs. 63%), but the difference was not statistically significant (Fisher exact test *p* = 0.571). Female respondents attended non-mandatory lectures at approximately the same rates as male respondents (65% vs. 56%; *p* = 0.391). The percent of students attending some or all non-mandatory lectures grouped by intended specialty varied from 44% (psychiatry) to 72% (pediatrics; Fig. 1), but the differences were small relative to the 95% confidence intervals. In general, students who preferred less competitive specialties attended lectures more often than those who were aiming for more competitive specialties.

### Why Don’t Students Go To Class?

**Fig. 2:**
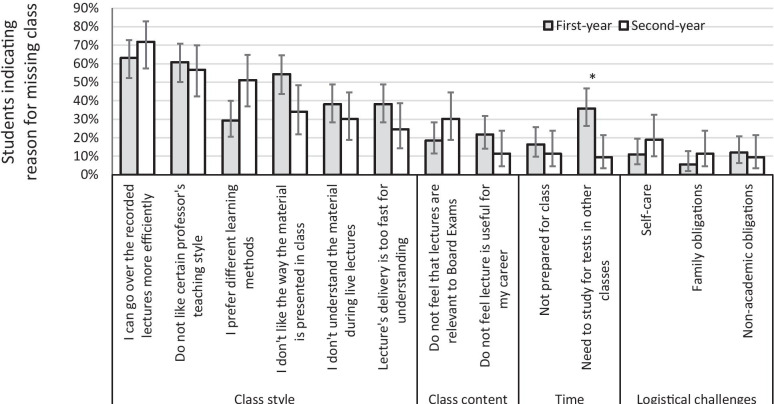
Percent (95% confidence intervals) for first-year and second-year) respondents regarding reasons to miss class. *Difference between first-year and second-year *p* ≤ 0.01

The class style was the most common area of reasons for students to miss class, followed by class content, time management, and logistical challenges (Fig. 2). The increased efficiency of viewing lectures online was the top reason for missing class in both cohorts, and having issues with professors’ teaching style was the second-most cited reason. In general, first and second-year students indicated similar reasons for missing class. One notable exception was that first-year students were more than three times more likely than second-year students to report they missed class because they needed to study for tests in other disciplines (Fig. 2). Students who provided additional reasons for missing class listed the efficiency of using recorded lectures, avoiding a commute, educational style, lecture content, and self-care, such as lack of sleep, getting exercise, eating, and reducing mental overload.

Learning style was not a strong indicator of whether a student would attend non-mandatory class sessions. Students who indicated that they learned best by using mnemonics had 2.5 increased odds of attending at least some non-mandatory class sessions, but none of the other learning styles were associated with lecture attendance (Table 2). The confidence that students expressed in the value of class in preparing them for Boards or evidence-based practice was also not predictive of non-mandatory class attendance (Table 2). However, only 15.2% and 22.1% stated they were confident or very confident, respectively, in medical school classes to prepare them for Board exams and evidence-based medicine.

**Table 2:** Odds ratios with 95% confidence intervals, likelihood chi-square statistics, degrees of freedom (*df*), and *p* value relating learning styles to non-mandatory class attendance

	Odds ratio	95% confidence interval		LR chi^2^	*df*	*p* value
Create flowcharts	1.46	0.64	3.36	0.80	1	0.370
Discuss material with others	1.66	0.76	3.65	1.64	1	0.201
Draw and paraphrase	1.26	0.53	2.97	0.28	1	0.596
Listen to recordings	0.58	0.26	1.26	1.91	1	0.167
Read the lecture slides	0.80	0.36	1.71	0.34	1	0.560
Read the textbooks	0.75	0.27	2.09	0.30	1	0.582
Spaced repetition	0.94	0.41	2.13	0.02	1	0.889
Study with a group of three to five people	1.01	0.34	3.16	0.00	1	0.984
Use flashcards and ANKI decks	0.73	0.32	1.64	0.56	1	0.452
Use mnemonics	2.47	1.09	5.74	4.67	1	0.031
Use visuals	0.89	0.39	2.00	0.07	1	0.786
Confidence that class prepares for Boards	1.21	0.94	1.55	2.26	1	0.133
Confidence that class prepares for EBP	1.02	0.79	1.33	0.03	1	0.857

### What Materials Do Students Use to Study Outside of Class?

**Fig. 3:**
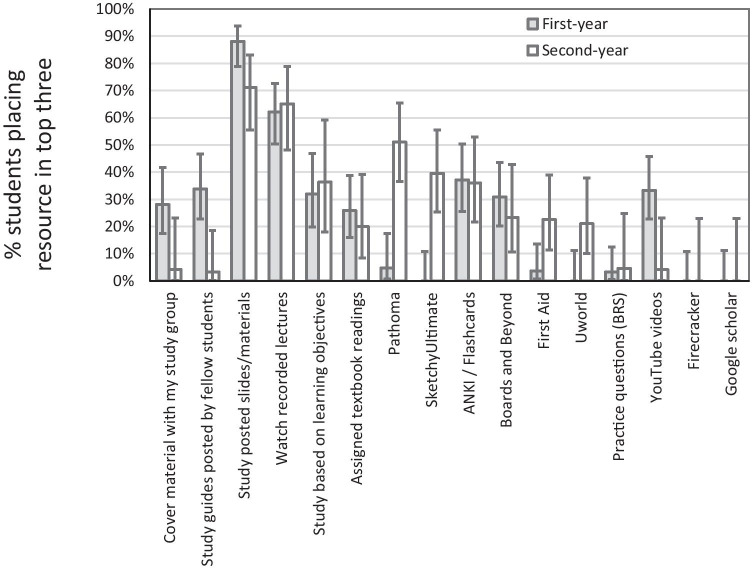
Percent (95% confidence intervals) for first-year and second-year respondents regarding resources used outside of class. Differences between first-year and second-year students were not directly compared in the statistical analysis

The most frequently used resources revealed some areas of agreement and some areas of dissent between first-year and second-year respondents. The most-used resources for respondents in both cohorts were slides provided by the faculty members and recorded lectures (Fig. 3). When the two cohorts disagreed, second-year students favored Pathoma, Sketchy Ultimate, First Aid, and UWorld, whereas first-year students favored YouTube videos, study guides posted by classmates, and covering material with a study group more than their second-year counterparts.

### What Would Motivate Students to Attend Class More Often?

**Fig. 4:**
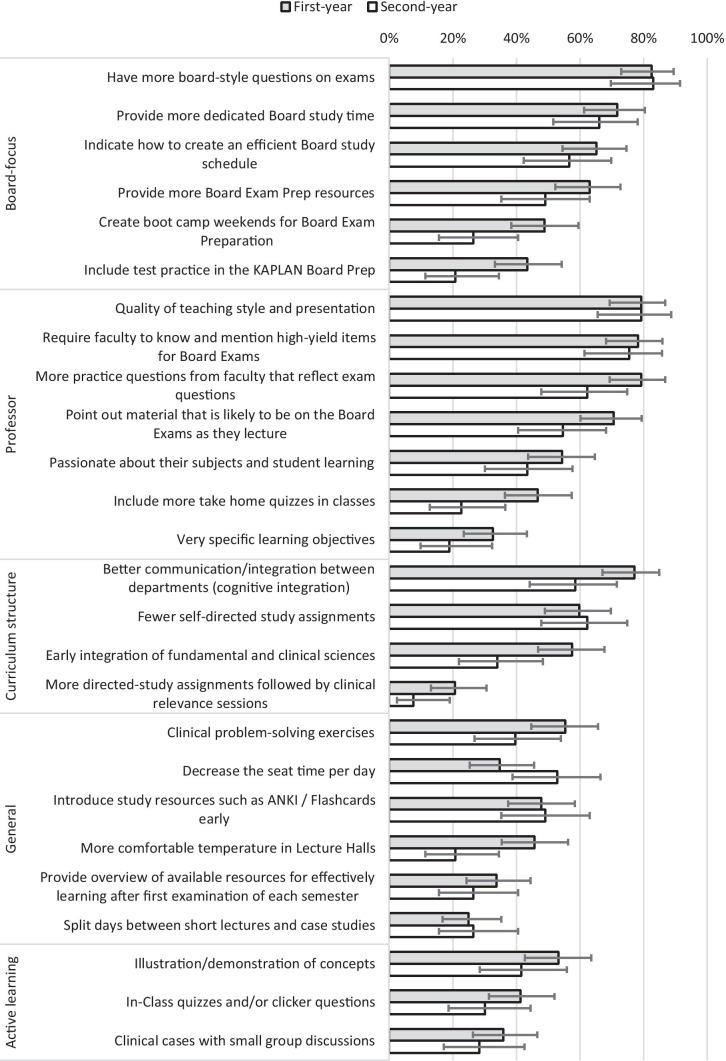
Percent (95% confidence intervals) for first-year and second-year respondents regarding changes that might encourage class attendance. Differences between first-year and second-year students were not statistically analyzed

Over 75% of respondents from both cohorts said they would attend class more if there were more Board-style questions on the summative exams, if the teaching style and presentation were changed, and if the faculty were required to know and mention high-yield items for Board exams (Fig. 4). The most selected choice for increasing attendance focused on increasing emphasis on Board exams and teaching decisions. Respondents then indicated that changes to the curriculum structure in the form of better cognitive integration among departments and fewer self-directed study assignments might also increase attendance. Less popular were responses related to active learning adjustments to in-class presentations, general changes, and logistics.

First-year students tended to be more optimistic regarding changes that could be made to motivate them to attend class, as most of the offered changes had a higher percentage of votes from first-year relative to second-year students (Fig. 3). The only intervention that second-year students supported notably more than first-year students was decreasing the seat time per day.

## Discussion

Poor attendance in the pre-clinical years is a global issue, which has sparked interest in its causes [1, 3, 11, 19, 20] and effects on students’ achievement [21]. However, few studies have asked students what would motivate them to resume attending live lectures. This study is the first to focus on student suggestions for improving attendance to help shape actionable strategies for medical educators. Our survey results are in agreement with other studies in reporting that students miss non-mandatory lectures due to issues with content delivery and inefficiencies of attending the lecture in person [7, 19]. Most importantly, the results of this study suggest that students’ concerns regarding competitiveness on Board exams may be the strongest underlying contributor to poor lecture attendance.

### Concerns About Competitiveness

The results from the survey indicate that students found the content of live class sessions to be useful. Ideally, class materials and activities should fill the dual role of fostering high grades on class examinations and increasing performance on Board exams. The results of this study suggest that students perceive the material presented in class as helpful with grades, but they wish it would be more aligned with Board preparation. For example, most students wanted more Board-style questions on exams, and they would like the faculty to emphasize high yield subjects for Board exams. This trend was more evident for second-year students who shift from a focus on study groups and other course-specific content to Board-specific materials. When asked, fewer than two in five students said they were confident that classes prepare them for Boards. It is also noteworthy that students who are targeting competitive residencies appear to skip class the most. The data of this survey corroborated the findings by Cardall et al. [22], whereby students’ decision to use video-recorded lectures instead of attending live lectures was primarily motivated by a focus on their professional goals. The perceived disconnection between classroom learning and extensive Board exam material might make students feel insecure about their ability to adequately prepare for Board exams. This concern might be misguided because students in this study did not have firsthand experience of the Board exams when they completed the survey.

Alignment between Board exams and curriculum at the level of individual faculty members requires deep understanding and engagement in the process of writing Board-style exam questions. If faculty neither make an effort to learn about the Board exams nor transmit that knowledge, the students' insecurities about Board exam preparation are not addressed. Making regular connections between Board exams and course materials would eliminate the sometimes false dichotomy between working towards high grades and working towards high Board scores. This approach may encourage students to value class time. In fact, little change in curriculum content may be required in many cases, although there is some evidence that assessment styles, at least, could be better aligned with Board exam expectations [23].

While Board exam scores are clearly important for residency placements, students must also be equipped with the knowledge and skills that will make them good physicians beyond their performance on those exams [24]. Certain traits such as professionalism, critical thinking, and evidence based practice of medicine can only be efficiently imparted to students in a classroom setting. Educators may have to rise to the challenge of incorporating materials students find useful to their goal of performing well on Board exams while creating an interactive classroom environment that will also make students look beyond licensure exams.

### Issues with Content Delivery

In this study, respondents indicated that class or professor style also discouraged attendance. It is possible that students with a particular learning style would prefer to attend lectures, but with the exception of students who preferred to learn using mnemonics, there was no support for the idea that learning style plays a key role in attending live lectures. This is surprising, as other work has indicated that learning style should be considered in developing approaches to medical training [25]. In the end, medical students value their learning time and must be convinced that time spent in a live lecture is superior to alternative uses of that time. Other studies have found that if students feel that current in-class practices are not an efficient use of their time [26], medical students may continue to “vote with their feet.”

Medical schools have a choice to adopt a traditional lecture model, a full problem-based learning model, or some combination of passive and active learning. A review of 43 articles concluded that medical students in pre-clinical years were strongly satisfied with active learning modalities that are learner-centered over the traditional lecture-based learning [27]. This conclusion should not group all lecturing modalities under the same umbrella. For example, the use of interactive PowerPoint-based lectures with clicker-based formative assessment [28] is not equivalent to passive oration discourse alone. Another disadvantage associated with lecturing is that students place a great emphasis on the professor’s preparation, presentation, and speaking style. In a large class, it is virtually impossible to adopt an approach that will be well received by all in the audience. The focus on lecturing by the teacher, who may not be a clinician, deprives the students of the clinical context. The traditional didactic lecture may be the preferred method for transferring foundational scientific medical knowledge, but without clinical scenarios to demonstrate its applicability, students may think the material is irrelevant to their future professional lives.

For more than a decade, studies have been emphasizing the importance of active learning in improving students' critical thinking skills, allowing for higher-order thinking, increasing class engagement, and demonstrating the applicability of the course content [29–31]. The results of this study were, therefore, surprising because students did not favor increased in-class active learning, such as illustrating concepts using clinical cases and concept mapping. There are several reasons why medical students might not favor active learning: (A) they are unfamiliar with active learning [32], (B) the faculty members are  not familiar with the learning the techniques [32], or (C) students are very focused on time spent to learn facts, and active learning seems inefficient.

In support of reason A, students in this study had some mandatory clinically integrated sessions (CIS), but they represent a very small proportion of the overall class time. If reason B is the case, resources are available to help instructors implement active learning successfully. For example, the International Association of Medical Science Educators has been offering focus sessions for many years so medical educators can move from being information givers to student-centered learning facilitators [31]. There are at least 25 active learning techniques that have been shown to work in large group settings [31]. It may suffice to try a few and select those that are more learning-centered.

### Learning Efficiency

The availability of video-recorded lectures provides students with the choice of staying home and still “attending” class. When live lectures were compared to viewing video-recorded lectures, the video-recorded lectures were found to be equally effective, as student performance was similar [33] or even more efficient for faster knowledge acquisition, thereby leaving time for other activities [22]. Every student has a different knowledge base and may have difficulty with different concepts. The capability of the video recording to speed through some topics, slow down for others, and allow replay an infinite number of times means that students are able to customize video lectures, which is not available for in-person lecture sessions. As highlighted above, there is some evidence that lecturing is not the most efficient way to deliver material in the first place [10, 20], so there may be a need to reconsider the types of content delivery. On the other hand, attending a lecture in real-time allows student access to the instructor for follow-up questions that can help resolve misunderstandings and allows instructors to feel they have an engaged audience.

The finding that students wanted more cognitive integration across the curriculum, a topic that has guided changes in undergraduate medical education at other institutions [34], may also be related to efficiency. If some material is repeated multiple times while other, equally important, material is covered in a cursory manner, students will avoid the classroom. They will instead review the video lecture, where repeat material can be quickly sped forward to avoid wasting time.

Medical students are tasked with managing their time in a way that allows maximum learning in minimum time, given the large quantity of information they are asked to absorb. The survey results suggest that some students, particularly in the first year, may still need to improve their time management skills. There are multiple ways that medical schools can help students develop better time management skills [35].

Responses to the survey also suggest that students with strong time management skills believe they are making a rational choice in skipping live lecture sessions. Attending a lecture session involves commuting and finding parking, so attending an hour-long lecture may involve two hours of time that could be spent in other ways. Medical schools can make the logistical costs of attending lectures lest onerous by blocking class sessions to minimize commutes.

### Limitations

The applicability of this study is limited in that it focused on a single site and included students who had not yet taken Board exams, yet it tackled a problem that is held in common with other medical schools worldwide. It would be a useful follow-up to survey the same students after Board exams to understand whether their apparent concern about preparation for Board exams relative to the class sessions were well-founded. Although the response rate was reasonably high, it is likely that the survey responses disproportionately represent students who attend class regularly due to a closer engagement with the medical school, although we know there was little gender bias in the sample. It may also be the case the respondents were more likely to be unhappy with some aspect of their schooling compared to non-respondents. A larger sample would have allowed a more complete analysis of the attendance data, since there may be differences among groups who always attend lectures and those who often or sometimes attend lectures.

## Conclusion

Our hypothesis that students would be motivated to attend class by more active learning approaches and better cognitive integration across the curriculum was only partially supported. The results of this study corroborate other work that has identified preparation for Board Examinations as the primary purpose (in students’ minds) of learning during the pre-clinical years of undergraduate medical education. Better alignment with and communication about Board exams during classes may help alleviate students’ concerns about competitiveness relating to these exams. One approach may be to require faculty to write objectives on high frequency and/or high impact topics and prepare Board-style questions, thereby insuring more efficient Board preparation. In addition, good teaching practices still matter as well. Instructors can make the most of live sessions with students through targeted faculty development to improve teaching practices. If teaching practices improve Board exam performance, these results should be clearly communicated to the students to help them have confidence that class sessions are valuable to their future.

## Supplementary Information

Below is the link to the electronic supplementary material.

Supplementary file1 (PDF 120 KB)
