# Supplementary material for: Study of Live Lecture Attendance, Student Perceptions and Expectations
Source: Med Sci Educ. 2021 Feb 23;31(2):697–707. doi: 10.1007/s40670-021-01236-8 (PMC8368907; doi:10.1007/s40670-021-01236-8)
Supplement: Supplementary file 1 — Supplementary file1 (PDF 120 KB) [file 40670_2021_1236_MOESM1_ESM.pdf]

Nevada

STUDY OF MEDICAL SCHOOL LACK OF LIVE LECTURE ATTENDANCE

CAUSES AND EXPECTATIONS

"Why DO Students NOT Attend?"

1. In the last three months, how often have you attended live lectures?

- a. Every day
- b. Attended most lectures (Skipped 1-3 lectures/week)
- c. Attended half of the lectures (Skipped 4+ lectures/week)
- d. Attended mandatory lectures only
- e. Other, explain:

2. When you do not attend class, what do you do instead to learn and prepare for the Summative Exams?

Select all that apply *in the order of importance*.

- a. ANKI / Flashcards
- b. Assigned textbook readings
- c. Boards and Beyond
- d. Cover material with my study group
- e. Create study guides based on learning objectives
- f. Firecracker
- g. First Aid
- h. Google scholar
- i. Pathoma
- j. Practice questions (BRS)
- k. SketchyUltimate
- l. Study guides posted by classmates and/or upperclassmen
- m. Study posted PowerPoints/Materials
- n. Uworld
- o. Watch recorded lectures
- p. YouTube videos

3. If you attend live lectures, what do you do before class to prepare?

Select all that apply.

- [ ] a. Preview the material briefly
- [ ] b. Preview the material extensively
- [ ] c. Preview using Board Exam Prep material
- [ ] d. Read assigned textbook chapters
- [ ] e. Watch videos/lectures from other sources
- [ ] f. Other, specify:

4. When you attend live lectures, which of the following do you do in class?

Select all that apply.

- [ ] a. Ask clarifying questions
- [ ] b. Pay attention, but take a few to no notes
- [ ] c. Take notes while in class
- [ ] d. Other, specify:

5. Provide your reasons for missing lectures.

Select all that apply.

- ☐ a. I can go over the recorded lectures more efficiently
- ☐ b. Not prepared for class
- ☐ c. Need to study for tests in other classes
- ☐ d. Do not feel lecture is useful for my career
- ☐ e. I don't like the way the material is presented in class
- ☐ f. Lecture's delivery is too fast for understanding
- ☐ g. I don't understand the material during live lectures
- ☐ h. I prefer different learning methods
- ☐ i. Do not feel that lectures are relevant to Board Exams
- ☐ j. Do not like certain professor's teaching style
- ☐ k. Family obligations
- ☐ l. Non-academic obligations
- ☐ m. Self-care, such as (specify below):
- ☐ n. Other, specify:

6. What would make you more likely to attend live non-mandatory lectures?

A. Professors

Select all that apply.

- ☐ Professors who are passionate about their subjects and student learning
- ☐ Professors who point out material that is likely to be on the Board Exams as they lecture
- ☐ Quality of Professor's teaching style and presentation
- ☐ Other, specify:

7. What would make you more likely to attend live non-mandatory lectures?

B. Structure of Learning Sessions

Select all that apply.

- ☐ Clinical Cases with small group discussions
- ☐ Clinical problem-solving exercises
- ☐ Decrease the seat time per day
- ☐ Illustration/demonstration of concepts
- ☐ In-Class quizzes and/or clicker questions to check for understanding
- ☐ Split every day between short lectures and case studies
- ☐ Very specific learning objective for every lecture and discipline
- ☐ Other, specify:

8. What would make you more likely to attend live non-mandatory lectures?

C. Curriculum Structure

Select all that apply.

- ☐ Better communication/integration between departments (cognitive integration)
- ☐ Early integration of fundamental and clinical sciences
- ☐ Fewer Self-Directed Study assignments
- ☐ More Directed-Study assignments followed by their clinical relevance sessions
- ☐ Other, specify:

9. What would make you more likely to attend live non-mandatory lectures?

D. Classroom Environment

Select all that apply.

- ☐ More comfortable temperature in Lecture Halls (often too cold or too hot)
- ☐ Other, specify

10. How confident are you that your classes are preparing you for Board Exams?

- a. Very Confident
- b. Confident
- c. Somewhat confident
- d. Not confident
- e. Cannot judge at this point

Other, specify:

11. How confident are you that classes are preparing you for evidence-based medicine?

- a. Very Confident
- b. Confident
- c. Somewhat confident
- d. Not confident
- e. Cannot judge at this point

Other, specify:

12. What can your institution do to better help you reach your goals?

Select all that apply.

- ☐ a. Provide an overview of available resources for effectively learning after the first examination of each semester
- ☐ b. Create boot camp weekends for Board Exam Preparation
- ☐ c. Early introduction to study resources such as ANKI / Flashcards
- ☐ d. Have more Board-style questions on Exams
  - i. NBOME question bank
  - ii. NBME question bank
- ☐ e. Include more take home quizzes in all systems
- ☐ f. Include test practice in the KAPLAN Board Prep
  - i. Half-length during first three (3) weeks
  - ii. Full-length during the last week
- ☐ g. Indicate how to create an efficient Board-study schedule
- ☐ h. Provide more Board Exam Prep resources
- ☐ i. Provide more dedicated Board-study time
- ☐ j. Request more practice questions from faculty that reflect Exam questions
- ☐ k. Require the faculty to know what is high-yield for Board Exams and mention it during lecture
- ☐ l. Other, specify:

13. As a medical student, how can you improve your study habits and preparation for tests (Exams and Board Exams)?

A. Overall:

Select all that apply.

- ☐ Identify my weaknesses early in the semester and ask for a tutor
- ☐ Read the textbook
- ☐ Use the resources and study tools provided by the institution
- ☐ Other, specify:

14. As a medical student, how can you improve your habits and preparation for tests (Exams and Board Exams)?

B. Before a class period:

Select all that apply.

- [ ] Review the content of the PPNT provided
- [ ] Review resources ahead of time
- [ ] Other, specify:

15. As a medical student, how can you improve your study habits and preparation for tests (Exams and Board Exams)?

C. During a class period:

Select all that apply.

- [ ] Ask questions about the content
- [ ] Actively participate in class discussion
- [ ] Actively participate in small group activities
- [ ] Attend live lectures
- [ ] Work through clicker questions
- [ ] Other, specify:

16. As a medical student, how can you improve your study habits and preparation for tests (Exams and Board Exams)?

D. After a class period:

Select all that apply.

- [ ] Discuss material that I do not understand with faculty
- [ ] Organize learning teams to teach, encourage, and assess each other
- [ ] Self-assess and fill in the gaps before Exams
- [ ] Write questions using the learning objective/outcomes
- [ ] Other, specify:

17. How do you learn best?

Select all that apply.

- ☐ a. Create flowcharts
- ☐ b. Discuss material with others
- ☐ c. Draw and paraphrase
- ☐ d. Listen to recordings
- ☐ e. Read the lecture slides
- ☐ f. Read the textbooks
- ☐ g. Spaced repetition
- ☐ h. Study with a group of three to five people
- ☐ i. Use flashcards and ANKI decks
- ☐ j. Use mnemonics
- ☐ k. Use visuals
- ☐ l. Other, specify:

18. When do you learn best?

Select all that apply.

- ☐ a. Morning
- ☐ b. Middle of the day
- ☐ c. Evening
- ☐ d. When I have time
- ☐ e. No preference
- ☐ f. Other, specify:

19. What helps you manage stress and avoid burnout?

Select all that apply.

- ☐ a. Board games/video games
- ☐ b. Exercise and physical activity
- ☐ c. Reading
- ☐ d. Spending time outdoors
- ☐ e. Spending time with friends and family
- ☐ f. Travel
- ☐ g. Watching TV and movies
- ☐ h. Social media
- ☐ i. Other, specify: ____________

Demographic Information

20. What is your current GPA?

- ◯ a. 2.0 - 2.5
- ◯ b. 2.5 - 3.0
- ◯ c. 3.0 - 3.5
- ◯ d. 3.5 - 4.0

21. What is your gender?

- ◯ a. Female
- ◯ b. Male

22. What specialties are you interested in?

Select and Rank your *TOP THREE*.

- [ ] a. Allergy & Immunology
- [ ] b. Anesthesiology
- [ ] c. Colon & Rectal Surgery

- d. Dermatology
- e. ENT-Otolaryngology
- f. Emergency Medicine
- g. Family Medicine
- h. Gastroenterology
- i. Geriatrics
- j. Hematology/Oncology
- k. Internal Medicine
- l. Medical Genetics and Genomics
- m. Neurological Surgery
- n. Nuclear Medicine
- o. Obstetrics & Gynecology
- p. Ophthalmology
- q. Orthopedic Surgery
- r. Pathology
- s. Pediatrics
- t. Physical Medicine & Rehabilitation
- u. Plastic Surgery
- v. Preventive Medicine
- w. Psychiatry
- x. Neurology
- y. Radiology

z. Surgery

aa. Thoracic Surgery

bb. Urology

cc. Unsure

23. What are your TOP TWO reasons for entering medicine?

a. Contribution to society

b. Family/Community Expectations

c. Helping others

d. High income

e. Professional prestige

f. Self-worth

g. Serve the medically indigent

24. Would you like to be entered to win a $200 Gift Card?

- No
- Yes, Enter your name below.

Thank you for your time, and thank you for your participation in this survey!
